# Small ruminant feed systems: perceptions and practices in the transitional zone of Ghana

**DOI:** 10.1186/1746-4269-6-11

**Published:** 2010-03-19

**Authors:** Stephanie Duku, Akke J van der Zijpp, Patricia Howard

**Affiliations:** 1 Wageningen University, Animal Production Systems Group, P.O. Box 338, 6700 AH Wageningen, The Netherlands; 2 Department of Crop and Soil Sciences, Kwame Nkrumah University of Science and Technology, Kumasi, Ghana; 3 Wageningen University, Social Sciences Group, Postbus 8130, 6700 EW Wageningen, The Netherlands; 4 Centre for Biocultural Diversity, University of Kent, Dept. of Anthropology, Marlowe Building, Canterbury, CT2 7NR, UK

## Abstract

**Background:**

Adequate feeding is essential to realizing the potential of small ruminants to alleviate poverty among smallholder farmers. This study was conducted in two villages in the Ejura-Sekyedumase District of Ghana and was motivated by farmers' non-adoption of modern feed technologies, but more importantly by the need to understand the small ruminant feed system considering farmers' different socio-economic backgrounds and how these relate to small ruminant performance. In this study, the feed system was defined as the type, source and seasonality of feeds and how small ruminants access them.

**Methods:**

Qualitative and quantitative methods were used to allow for triangulation. Data were collected in seven stages comprising key informant interviews, a census, a cultural domain study, botanical specimen collection and identification, focus group discussions, a household survey, and a small ruminant performance study.

**Results:**

Farmers listed 175 items that are used as small ruminant feed and salience indexes were calculated. There was high consensus about the domain of small ruminant feeds, with 15 items comprising the consensus model. Respondent agreement scores correlated positively with age and negatively with list length. Respondents from matrilineal lineages had higher agreement scores than those from patrilineal lineages. Natural pasture and wild browse scored high in pair wise ranking by village and sex groups. Of the 33 feeds that farmers fed to goats, maize grains, cassava peels and *Margaritaria discoidea *were the most salient. Six major feed system groups based on access were identified at household level, which regrouped into three at village level based on feed type and source. Patrilineal households were more likely to tether their livestock. Significant differences were found between some socio-economic groups for pre-weaning average daily gain (ADG) of kids, but not for prolificacy of does.

**Conclusions:**

The need for nutritive and agronomic investigations into major feeds, the creation of non-cropping zones around village fringes and studies on labour demands of different feed systems are proposed. The insight gained in this study on farmers' perceptions and practices relating to small ruminant feeds could guide in the selection and introduction of feed innovations that fit into current feed systems to enhance adoption.

## Background

Research has documented the potential of small ruminants for poverty alleviation [1-4]. Poverty levels in Ghana are highest among smallholder food crop farmers, with women farmers over-represented [5]. In the transitional zone, which has been labelled the breadbasket of Ghana [6], food crop farming is the major and minor occupation of 36% and 13% of all household members, respectively. Small ruminants are the major livestock species reared by smallholder crop farmers in this zone [7], which could be a means of alleviating poverty among these farmers, especially women and other vulnerable groups.

To increase the production of small ruminants profitably, adequate feeding is recognized as the most important factor, next to health [8]. In traditional systems with minimal cash inputs, small ruminant rearing mostly relies on family labour, most of which goes into grazing, herding or fodder collection [1]. A clearer assessment of the current feed situation in the transitional zone of Ghana is required if feeding is to be used as a basis for enhanced small ruminant production.

It has been claimed that the zone abounds in feed [9] and that small ruminants depend mainly on natural pasture and crop residue [7], though a decrease in grazing land and biodiversity attributed to the expansion of cropping areas [10] and feed shortages exacerbated by indiscriminate bush fires have also been reported [9]. Technologies such as urea treatment of straw, hay/silage making, pasture development and fodder bank establishment, promoted by the Ministry of Food and Agriculture (MOFA) extension agents and Non-Governmental Organizations (NGOs) to enhance feeding of ruminants, have had adoption rates of 2.8%, 0%, 1.4% and 2.8%, respectively, in the zone. Non-adoption of modern feed technology has been blamed on top-down approaches that do not take farmers' knowledge, circumstances and local technology into consideration [11,12]. Traditional technologies have evolved under specific cultural and environmental conditions and may therefore be seen to be culturally appropriate, locally available, inexpensive, and effective [11,12].

To identify the potential of small ruminant rearing for poverty alleviation in the transitional zone through adequate feeding, existing feeding practices in crop-livestock systems and farmers' knowledge and perceptions about feeds and feeding practices should first be sought, especially in the midst of rapidly changing ecological, social and cultural conditions [13]. Pioneering work in Ghana [14-16] has catalogued many species, their occurrence, biology and uses, some of which include the feeding of small ruminants. There is, however, a dearth of documented information regarding what farmers themselves collectively perceive as "feed for small ruminants" in the transitional zone. Moreover, there is no documentation regarding the relative importance of these feeds to farmers in the zone, although some researchers reported on feeds eaten by small ruminants in parts of the zone [17,18]. There is also a dearth of information on the modalities of feed usage by farmers in the zone, with respect to who uses which feed, feed sources, how different feeds are used and the seasonality of usage.

Farmers' knowledge is, however, not evenly distributed. It is recognised that socio-economic factors such as age, sex, religious affiliation, wealth, kinship, subsistence strategy, and individual competency result in differences in knowledge due to differential access to, use of, and familiarity with resources [[11,13,19], and [20]]. Howard [19] has defined gendered knowledge as "that which is held either by men or by women, but not by both". Her definition would imply a gender division of labour with respect to the use, management and conservation of plants as a reflection of gendered knowledge based on experience and practice. She argues further however that there is more to gendered knowledge than gender division of labour. For instance, men and women may use different spaces or use the same spaces differently. Moreover, women and men relate differently to different groups of people, leading to different social and knowledge networks and have different access to formal and exogenous knowledge [19]. Simpson's study in Mali [20] showed that women and men may not only possess knowledge of different things but different knowledge on similar things as well. In addition to gender differences in indigenous botanical knowledge, Ayantunde et al. [13] found significant ethnic and age differences in botanical knowledge. Howard [19] argues that there is a relationship between plant knowledge, power and social status. A close relationship between livestock, religion, and culture was also reported [21].

The transitional zone of Ghana continues to experience an influx of migrants, especially from northern parts of Ghana, to engage in farming and other activities [22,23]. The zone is thus ethnically diverse, with people of different socio-economic backgrounds, which could have an impact on knowledge distribution. Some studies have catalogued the interconnections between socio-economic factors and crop production in the zone [22,23]. With respect to small ruminant production, little is known about the linkages between socio-economic factors and the feed system and how these relate to animal performance.

The overall objective of this study was, therefore, to understand the linkages between the small ruminant feed system, farmers' socio-economic circumstances and small ruminant performance. The specific objectives were:

• To identify and document what farmers generally classify as small ruminant feeds

• To classify the small ruminant feed system

• To investigate relationships between the small ruminant feed system, farmers' socio-economic circumstances and small ruminant performance.

## Methods

### Study area

**Figure 1:**
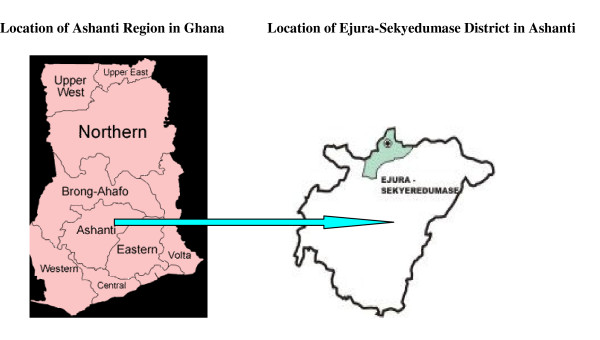
Map of the Ejura-Sekyedumase District of the Ashanti Region, Ghana

The study was undertaken in the Ejura-Sekyedumase District of the Ashanti Region of Ghana (Figure 1). The district is ethnically heterogeneous with a high concentration of smallholder crop farmers, considered nationwide as the occupational group with the highest incidence of poverty. The population is 81,115, out of which 52% are males and 48% are females. The district lies within longitudes 1°5' W and 1°39' W and latitudes 7°9' N and 7°36' N, covering an area of 1,782.2 km^2^. It has a bimodal rainfall pattern ranging between 1200 and 1500 mm with a major rainy season from April to August, and a minor rainy season from August to November. The district experiences both forest and savannah climatic conditions with both forest and savannah vegetation (Unpublished data: Ejura-Sekyedumase District Profile).

The major crops such as maize, cowpea, groundnuts, rice, cassava, yam, garden egg, pepper, and okra are produced mostly for sale. Some farmers cultivate tree and agro-forestry crops such as cashew, mango, and teak. Livestock species kept are cattle, sheep, goats, poultry, a few pigs, and non-traditional species (grasscutter, snails, and bees). About 60% of livestock farmers keep small ruminants. Of the small ruminant farmers, about 60% keep goats, 80% practice free range management and 65% do not provide housing for their stock (Nyarko, Senior Animal Husbandry Officer, MOFA, Ejura - personal communication). Respondents in a study by MOFA in 2008 considered that about 60% of livestock in the district are small ruminants, with natural pasture, shrubs, and crop peels as the major feeds.

Within the district, two villages, Kasei and Kobriti, were purposively selected after a mini census was carried out during a reconnaissance study of the district. The selection criteria used were: location in the transitional zone, rural but accessible with a sufficient number of small ruminant-keeping households to allow for comparison between village, sex, lineage, religious and economic status groups, and which were also willing to take part in the study. Kasei and Kobriti had populations of about 1446 and 388, respectively, at the beginning of the study. The former has a hospital, primary and junior high schools, a small market, and piped water which rarely flows. The latter has a primary school and a water borehole as the only infrastructure and has denser vegetation, being on the fringe of the transitional zone.

### Data collection

For the purpose of triangulation [24], both quantitative and qualitative methods were used to collect data in seven stages namely: key informant interviews, a census, a cultural domain study, botanical specimen collection and identification, focus group discussions, a household survey, and a small ruminant performance study.

#### Key informant interviews

Key informants were selected first, using snowball sampling, starting with extension agents who guided the selection of other key informants who were considered to have good knowledge of specific issues of interest to the study and were prepared to share it [25]. Key informants gave insights into the ethnic, religious, and socio-economic composition of the communities, crop and livestock farming practices, land tenure systems, and gender issues. Information obtained from key informants also contributed to the refinement of the survey questionnaire. Interviews were conducted in March and April 2007 with 11 informants aged 32 to 76 years, six of whom were male and five of whom were female, using a semi-structured questionnaire tailored to suit each informant. Audio recordings of key informant interviews were transcribed verbatim.

#### Census

Next, a structured questionnaire was administered to the heads of all the 407 households in the selected communities on demographics of household members, crop acreages in the previous year (2006), presence and number of small ruminants, and years of experience in small ruminant rearing. The census aided in the selection of freelisting and focus group participants, allowing representation of different socio-economic groups, and of household survey respondents. The census showed that 30% of households in the two villages kept small ruminants, with three per cent having only sheep, 19% having only goats and eight per cent with both sheep and goats. Thus, 90% of small ruminant keeping households had goats. With regards to feeds fed, farmers said that the same feeds were used for sheep and goats. Based upon this, only households with goats were selected for further research in order to obtain a representative sample for further study on feeds and performance.

#### Cultural domain study

Cultural domain analysis is used to ascertain whether people from a particular culture recognise a particular category of phenomena (e.g. 'wild foods', 'small game animals'), and which items pertain to that domain. In this study, freelisting [25,26] was used to determine whether the cultural domain of 'small ruminant feeds' exists, and whether there is consensus among farmers about what constitutes the domain and about the relative importance of each feed within the domain. Farmers were asked to mention all the 'small ruminant feeds' they knew and these were listed in the order given. In cultural domain analysis, it is considered that the higher an item is on the list, the more salient it is to the informant. Freelists were collected from 22 men and 19 women aged 20 to 75 years, who were selected by stratified random sampling to include all age and socio-economic groups.

#### Botanical collection

Next, voucher specimens of the freelisted species that the researcher could not easily identify were collected with the assistance of farmers. Farmers who mentioned the species were consulted when the need arose. The species were labelled with their local names, pressed, dried, and sent to the Forestry Research Institute of Ghana and the Botany Department of the University of Ghana for mounting and identification.

#### Focus group discussions

One male and one female focus group were created for each village for free and optimal expression of opinion by each sex. The groups comprised mostly of the freelisting exercise participants, and were the sources of data for village Forage Resource Maps, Landscape Niche Calendars, and a Feed Rank Matrix. Howard and Smith's [27] methods were used for the Forage Resource Maps and Landscape Niche Calendars. For the former, important landmarks in each village such as roads, churches, and schools were plotted for initial orientation, and major feed locations were added later. These maps indicated the proximity of forage sources to homesteads. Landscape niche calendars revealed the seasonal availability of feeds and niche use. Feed matrix ranking was used to elicit feed preferences of focus group participants and their motivations for using them. Audio recordings of discussions were transcribed verbatim.

#### Household survey

A household survey was carried out to collect household information on feed types, sources, access by small ruminants and seasonality of access. Households were selected by stratifying census data by ethnicity, religion, household headship, socio-economic status and the presence of small ruminants. Female headed households were purposively selected due to small numbers. The variables placed households in different contexts in terms of cultural norms, access to and control over resources, and roles and responsibilities, which could influence their choices with respect to feeds and feeding [4,13,19]. Twenty three male and 13 female headed small ruminant-rearing households were selected from matrilineal Christian Akan, patrilineal Christian Gurma, and patrilineal Moslem Moshi groups. Economic status was the next criterion considered, and households with heads of low, middle and high economic status were selected for purpose of comparison, using maize acreage as proxy for wealth status (Nyarko, Senior Animal Husbandry Officer, MOFA, Ejura - personal communication).

#### Small ruminant performance study

Finally, a small ruminant performance study was carried out to explore relationships between the performance of West African dwarf goats and the feed system, with average daily gain and prolificacy as performance measures. Seventeen male and eight female headed households were initially selected for the study but some did not show commitment. In the end, pre-weaning weights (birth - 3 months) of 37 kids from six male-headed and three female headed households were monitored between April and August, 2008. The number of kids dropped by 58 mature does from nine male headed and five female headed households were obtained by farmer recall up to previous three parities.

### Data analysis

Freelist data were analysed using the ANTHROPAC programme [28] to calculate the frequency and salience (Smith's S) of feeds. Salience is a measure of the average rank of an item across all farmers' lists, weighted by the length of the lists in which the item occurs [29]. Freelists were also subjected to consensus analysis, which is a minimum residual factor analysis [30,31], using the ANTHROPAC programme [28], to establish the existence of a domain of small ruminant feeds, and to determine each informant's level of agreement with others on domain membership. A Pearson correlation was used to find the relationship between an informant's age, list length, and his or her agreement score (i.e. level of agreement with other informants). The list was subsequently grouped into feed categories - mainly natural pasture, cultivated multipurpose trees and shrubs (CMTS), wild browse, crops, crop residue, and crop by-products, using SPSS version 15 for Windows to generate descriptive statistics. In this study, crop residue refers to crop parts that are not usually harvested for food, and crop by-products are materials that remain after some crop processing. Transcribed audio recordings of key informant interviews and focus group discussions were analysed manually. Socio-economic variables used in analysis were village (Kasei, Kobriti), household headship (male headed, female headed), lineage (matrilineal, patrilineal), religion (non-Moslem, Moslem) and economic status (this was regrouped into lower and higher to facilitate data analysis).

Household survey data was analysed with SPSS (ibid). Cross tabulation of feeds fed against the source, access by small ruminants, and seasonal availability was done to identify feed system types at the household level. Feed system types were regrouped manually to identify feed systems at the village level. Likelihood ratio chi square was used to test significant differences for categorical variables due to the small dataset [32]. The Mann-Whitney test and One-way ANOVA were used to find differences in continuous attributes within socio-economic groups. Kid weights were analysed with Microsoft Excel to calculate pre-weaning average daily gain (ADG) separately for male and female kids. Prolificacy was calculated as the percentage of all kids dropped of all kidding. Mean ADG and prolificacy values were introduced as variables in SPSS and differences between categories of socio-economic variables within feed system types were explored using a t test.

## Results

### What farmers regard as small ruminant feed

**Figure 2:**
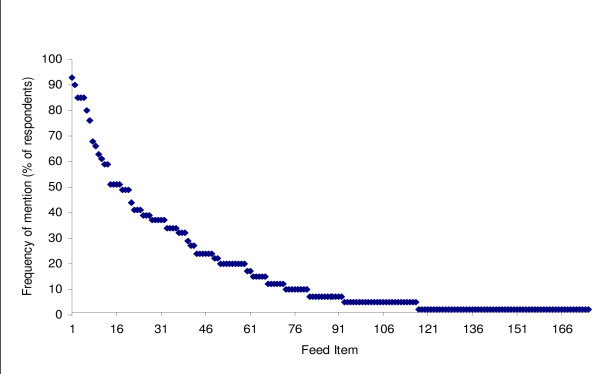
Sorted frequency of items in the domain of small ruminant feeds

There were a total of 175 items that the farmers who participated listed as small ruminant feed, belonging to 43 families, 105 genera, and 120 species, with three unclassified items (Additional file 1). Men free listed 145 items and women, 134. A total of 104 items were mentioned by both men and women. Freelist analysis yielded the frequency of mention of each item, its salience for all farmers, as well as for men and women farmers separately, and respondent-to-group comparisons. Figure 2 shows the relationship between items and frequency of mention.

Smith's salience indexes for the 15 items of the consensus model for all farmers (i.e. what all farmers agree on as small ruminant feeds), and the corresponding indexes for men and women are presented in Table 1. Smith's salience indexes fell progressively for all farmers, but not consistently for men and women. The most salient item for all farmers was maize grains. Items were not of equal salience to men and women. All peels and five out of seven crop residues were of higher salience to men than women.

**Table 1:** Smith's salience indexes (Smith's S) for the 15 members of the consensus model of small ruminant feeds.

ITEM	Salience for all farmers	Salience for men	Salience for women
Maize grains	0.667	0.613	0.731

Plantain leaves	0.629	0.647	0.610

Cassava leaves	0.609	0.559	0.667

Mango leaves	0.595	0.609	0.578

Cassava peels	0.586	0.685	0.472

Maize leaves	0.513	0.458	0.576

*Margaritaria discoidea*	0.467	0.534	0.390

Plantain peels	0.467	0.563	0.355

Cowpea leaves	0.466	0.519	0.405

Cassava tubers	0.426	0.414	0.439

Groundnut leaves	0.386	0.441	0.323

Yam peels	0.277	0.382	0.155

Baobab leaves	0.242	0.212	0.277

Palm leaves	0.230	0.246	0.212

Okra leaves	0.206	0.214	0.197

**Figure 3:**
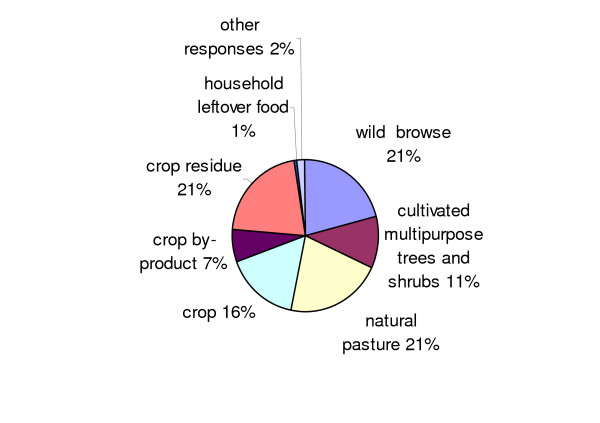
**Categories of freelisted small ruminant feeds in the transitional zone of Ghana**.

Consensus analysis (eigen value, 19.89; pseudo-reliability, 0.983) also compared individual freelists to the consensus model. Mean (sd) age (years), list length, and agreement score of the 41 individuals who participated in the freelisting exercise were 45.2 (15.2), 30.8 (10.2), and 0.8 (0.07), respectively. The Pearson correlation was positive between age and respondent agreement score (r = 0.339, p < 0.05), negative for list length and agreement score (r = -0.833, p < 0.01), but non-significant for list length and age. Informants from matrilineal lineages had significantly higher agreement scores on types of small ruminant feeds (Md = 0.82) than those from patrilineal lineages (Md = 0.77) (p = 0.05). No significant differences were found in agreement scores within all other socio-economic groups. The categorisation of freelisted items into feed groups (Figure 3) showed more items within the crop residue, natural pasture, and wild browse categories.

In the pair wise ranking exercise carried out with male and female focus groups which ranked feeds according to use by small ruminants, natural pasture species scored highest in both villages and among both sexes. There were differences in scores between Kasei and Kobriti for wild browse (10 vs. 6), between women and men for crop by-products (9 vs. 6) and for wild browse (9 vs. 7). Crops had no score in all groups.

### The small ruminant feed system

**Figure 4:**
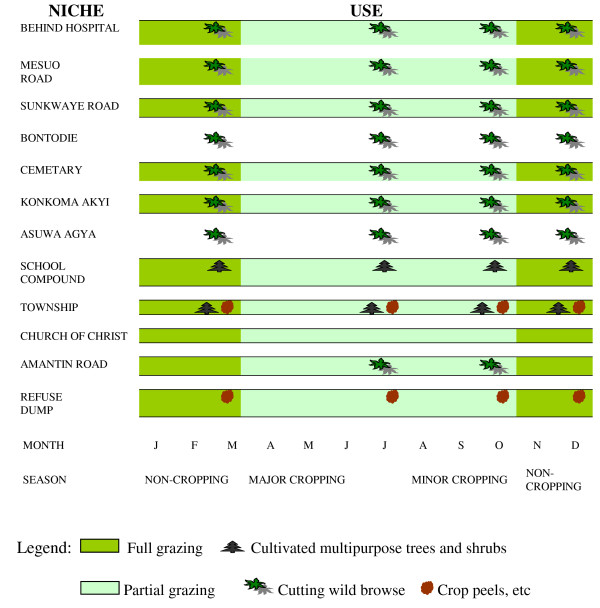
Landscape Niche Calendar -- Kasei

**Figure 5:**
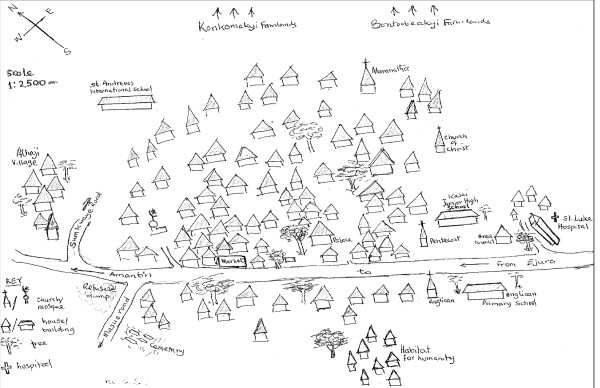
Forage Resource Map -- Kasei

**Figure 6:**
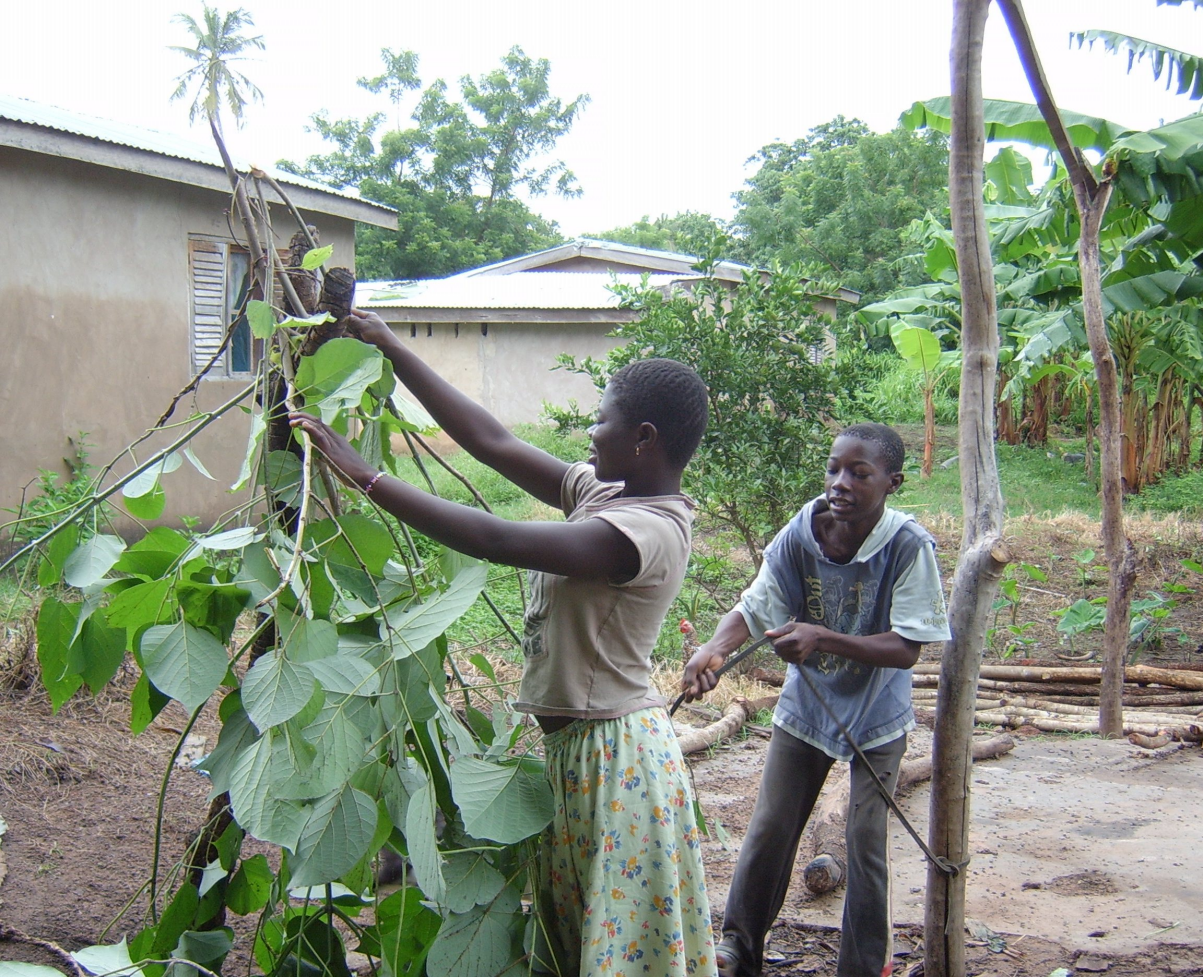
Children hanging feed for small ruminants at the backyard

Community level data were used to generate Landscape Niche Calendars and Forage Resource Maps. Twelve landscape niches were mentioned for Kasei (Figure 4). Six of these niches (behind the hospital, cemetery, school compound, township, Church of Christ, and refuse dump) were public places, while four niches (Mesuo road, Sunkwaye road, Konkomakyi, and Amantin road) were on privately owned lands on the village outskirts). These ten niches were used for scavenging and full grazing in non-cropping seasons and partial grazing in cropping seasons. The school compound and township were sources of CMTS for cut-and carry in all seasons (Figure 6) and wild browse was obtained from village outskirts. The township and refuse dump were sources of crop peels and other crop by-products. Bontodie and Asuwagya were more distant private farmlands used for cut-and-carry. Figure 5 shows the distribution of most landscape niches at Kasei. At Kobriti, all eight niches were used for grazing all year round with the exception of two, where grazing was restricted in cropping seasons. Wild browse was obtained from most locations and CMTS and crop peels were obtained from the township.

At household level, 36 heads mentioned thirty three feeds they themselves fed to goats (range, 2 - 11 feeds per household). Table 2 gives the Smith's salience indexes for the seven most salient items from freelist analysis of feeds fed, with comparative salience of feeds across four socio-economic groups. Maize grain, cassava peels and *Margaritaria discoidea *belonged to the consensus model in all socio-economic categories except the females group in which *M. discoidea *was absent. There was variation in other consensus items of different groups. Yam peels belonged to the consensus model in the female, Kasei and matrilineal groups, *Ficus umbellata *to the Kasei, male and higher status groups, and banana leaves to the higher status group. Females had higher salience indexes for cassava and yam peels than males.

**Table 2:** Smith's salience indexes (Smith's S) for seven most salient fed small ruminant feeds for farmer categories

Feed item		Smith's salience (S)							
	
	All farmers	Village		Sex		Lineage		Status	
		
		Kasei	Kobriti	Male	Female	Patri	Matri	Higher	Lower
Maize grain	0.678*	0.796*	0.441*	0.729*	0.586*	0.607*	0.766*	0.660*	0.695*

Cassava peels	0.468*	0.444*	0.514*	0.414*	0.562*	0.427*	0.518*	0.454*	0.481*

*Margaritaria discoidea*	0.422*	0.381*	0.503*	0.466*	0.342	0.426*	0.416*	0.479*	0.364*

*Ficus umbellata*	0.261	0.312*	0.159	0.252*	0.276	0.261	0.261	0.340*	0.182

Banana leaves	0.207	0.28	-	0.245	0.139	0.123	0.312	0.328*	0.086

Cassava leaves	0.152	0.104	0.25	0.154	0.15	0.133	0.177	0.113	0.191

Yam peels	0.145	0.181*	0.072	0.108	0.209*	0.107	0.192*	0.167	0.115

* Items with an asterisk belong to the consensus model of feeds fed within the group represented by the column

- The feed item in the row was not mentioned by the farmer category represented in the column

Feeds fed in 36 households are grouped into feed categories, by frequency of mention, in Table 3. Crop by-products had the highest frequency, followed by crops, wild browse, CMTS, crop residues, and natural pasture, in that order.

**Table 3:** Categorization of feeds fed and their frequencies of mention in 36 households

Feed category	Frequency of mention of feeds in category	Feed types
Wild browse	42	*Margaritaria discoidea*, *Pterocarpus erinaceus*, *Ficus sur*, *Ficus exasperata*, *Bridelia micrantha*, *Adansonia digitata*

Natural pasture	12	*Sida acuta*, *Andropogon gayanus*, *Pennisetum purpureum*, centro, *Digitaria insularis, Panicum maximum*

Cultivated multipurpose trees and shrubs	35	*Ficus umbellata*, *Gmelina arborea*, *Mangifera indica*, *Ficus sycomorus*, *Leucaena leucocephala*

Crop residue	34	Banana leaves, cassava leaves, plantain leaves, palm leaves, maize leaves, cowpea leaves, groundnut tops

Crop by-products	65	Cassava peels, yam peels, household food waste, maize flour, plantain peels, cowpea husk

Crops	44	Maize grains, cassava tubers, cowpea grains.

Total	232*	

* Not all 33 feeds were fed in all 36 households. This value represents the sum of frequencies for all feeds across all households. It is the number of household-feed cases.

**Figure 7:**
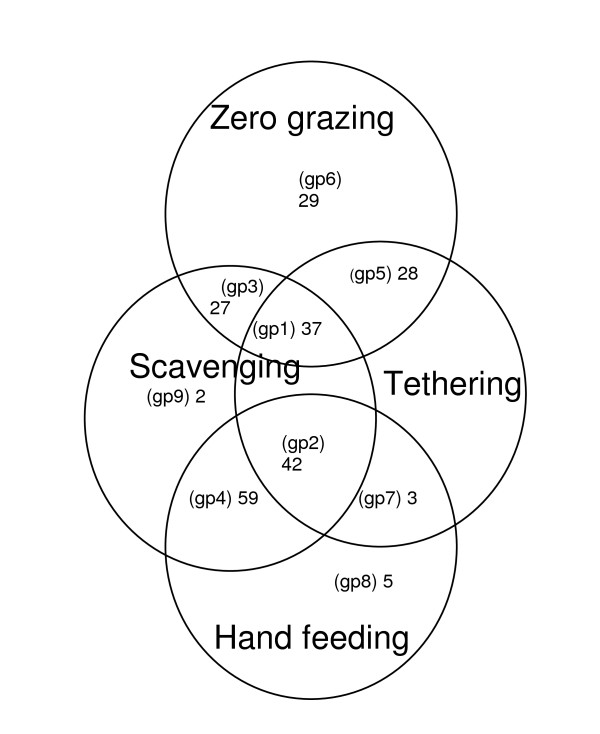
Venn diagram of access variables showing frequencies of household-feed cases in access combinations (access groups)

In subsequent analyses, 232 household-feed combinations were used, each constituting one case, with each case obtained from at least one source, accessed by goats in one or more ways, and available in a particular period of the year. Cross tabulations of feed, source, access, and seasonality variables showed that each case fell into a distinct group (access group) defined by a combination of access types (Figure 7), with no feeds accessed solely by tethering. There were six major (1-6) and three minor (7-9) groups. Description of major groups with frequency of cases and distribution of dominant cases (feeds) across sources and seasons is shown in Additional file 2.

Major feeds in Group 1, *Ficus umbellata*, banana leaves, and mango leaves, are leafy, accessed by goats through both tethering and zero grazing and by scavenging, and obtained from public lands, other people's private lands, and farmers' own home gardens, in all seasons. *Ficus umbellata *and banana are usually planted in the home garden but mango trees may or may not have been planted by the farmer him/herself. The major feed in Group 3 was *Ficus umbellata*, accessed by scavenging and zero grazing but without tethering, available in all seasons and obtained from the same sources as in Group 1 (Additional file 2).

Major feeds in Group 2 were maize grains, cassava peels and yam peels, in the category of crops and crop by-products and were accessed by both tethering and hand feeding and by scavenging. Maize grains are primarily from farmers' own production and peels were mainly from processing of farmers' produce for cooking, and to some extent from other people's kitchens. All of the feeds were available in all seasons, but maize was available to some farmers after the cropping season. Group 4 is similar to Group 2, but without tethering (Additional file 2).

Examination of access groups (Additional file 2) showed a pattern reflecting the existence of new groups, with 1 and 3 consisting of leafy feeds obtained mostly at the homestead or in the township, leafy feeds obtained mostly on farmlands in group 5 and crops and crop by-products in groups 2, 4, and 6. These new groups have been labelled 'leafyhome', 'leafyfarm' and 'cropnbyprod' respectively in Table 4, with a description in terms of source, access and season, and major feeds. Allocation of new groups to households showed that all 36 households belonged to the cropnbyprod group, and 35 also belonged to either or both of the leafy groups. A chi-square test for goodness-of-fit showed a significant difference in the proportion of households in leafy groups (p < 0.001).

**Table 4:** Regrouping of access groups based on source, access and season

New group	Composition (access groups)	Group description	Major feeds
Leafyhome	1,3	Obtained from homestead and township, accessed mostly by zero grazing with or without tethering, in all seasons	*Ficus umbellata*, Banana leaves, Mango leaves

Leafyfarm	5, 6	Obtained from farmlands, accessed by zero grazing with or without tethering but not by scavenging, used in cropping season by some households and all seasons by others	*Margaritaria discoidea*, *Pterocarpus erinaceus*, Cassava leaves

Cropnbyprod	2, 4	Crops and by-products obtained mostly from kitchen and accessed by hand feeding with or without tethering, mostly also scavenged and obtained in all seasons.	Maize grains, Cassava peels, Yam peels, Plantain peels, Cassava tubers

### Relationships between the small ruminant feed system, farmers' socio-economic circumstances and small ruminant performance

There was a significant association between lineage and most access groups, village and Group 6, and economic status and Group 1 (Table 5). All other socio-economic variables showed no significant relationships with access groups. Significant differences were found between matrilineal and patrilineal households (p ≤ 0.05) in tethering duration (12 vs. 9 hours) and age of household head (54.5 vs. 43 years) within some access groups. Matrilineal household heads in non-tethering access groups were older compared to patrilineal heads, and those that tethered, tethered longer.

**Table 5:** Significant relationships between access groups and socio-economic variables

Group		N	%	N	%	*X* ^2^	p
		
		Lineage					
		
		Matrilineal		Patrilineal			
1	Yes	5	31	13	65		
	
	No	11	69	7	35	4.05	0.04

2	Yes	5	31	14	70		
	
	No	11	69	6	30	5.36	0.02

3	Yes	10	62	4	20		
	
	No	6	38	16	80	6.76	0.01

4	Yes	14	87	8	40		
	
	No	2	13	12	60	8.44	0.00

5	Yes	4	25	14	70		
	
	No	12	75	6	30	7.20	0.01

		**Village**					
	
		**Kasei**		**Kobriti**			

6	Yes	6	25	7	58		
	
	No	18	75	5	42	3.85	0.05

		**Economic status**					

		**Lower**		**Higher**			

1	Yes	6	33	12	68		
	
	No	12	68	6	33	4.00	0.04

A Chi-square test showed a significant association between village group and leafy category group (p = 0.05). A post hoc test showed that households depending solely on leafy feeds obtained at the homestead were from Kasei. All other socio-economic variables showed no significant relationships with leafy groups. A one-way between-group ANOVA found no significant differences in household size, age of the household head, number of goats owned, and scavenging and tethering duration between groups.

For households obtaining leafy feeds from both homestead and farm, pre-weaning ADG was significantly higher for male headed than female headed households (39.9 g vs. 17.2 g; p < 0.05), for matrilineal than patrilineal households (40.2 g vs. 26.7 g; p < 0.1) and at Kasei than at Kobriti (37.8 vs. 23.8; p < 0.1). Religion and economic status had no significant effects on ADG. Prolificacy was neither significantly different between all socio-economic groups nor for households depending on leafy feeds from home and farm sources. Mean prolificacy across all households was 171%.

## Discussion

### What farmers regard as small ruminant feeds

The 175 items freelisted as small ruminant feed, belonging to 120 species, compare well with the 123 species collected by Ayantunde et al. [13], despite differences in method used and purpose. Their emphasis was on herbaceous and woody species in five major use categories one of which was forage. Moreover, they collected the species for farmers to identify, which could aid recall and identification. The freelisting method used in the present study has the advantage of allowing farmers themselves to name small ruminant feeds [31], which is a better indication of farmers' level of consciousness about what constitute small ruminant feeds. The few items that are mentioned by many respondents (Figure 1), being typical of freelists [33], are further reduced to the 15 items of the consensus model, which are the items more familiar to farmers, and where more farmers agree that they are small ruminant feeds (eigen value, 19.89; pseudo-reliability, 0.983). These items, being the most salient (Table 1), can be regarded as those most important and most likely to be used.

The individual agreement scores estimated by consensus analysis indicate how close to the consensus each individual's responses fall. High values indicate high agreement, while low values indicate that there is less agreement of the individual with a typical member of the group on what constitutes the domain of small ruminant feeds. The longer a list, the higher the tendency to mention many other items not mentioned by other farmers, resulting in the negative correlation between list length and agreement score. The positive correlation between respondent age and agreement score means that older members of the community are likely to agree more on what is generally considered as small ruminant feed, compared with younger members.

Significantly higher scores on the part of farmers from matrilineal lineage groups could be due to the longer exposure to items that were generally agreed on as small ruminant feed, since these farmers are indigenous to the district. This finding is comparable to that of Ayantunde et al. [13] who found a significant effect of ethnicity on recognition of herbaceous forage species, due to differences in exposure to such species between the Peulh ethnic group who were pastoralists and the Djerma who were land cultivators by tradition. The lack of significant differences in agreement scores within village, gender, religious, and status groups in our study, gives the impression of a general agreement on "what small ruminants eat". This impression is reflected in the assertion by focus groups and key informants that there are no differences in feeds fed by all categories of farmers. Thus, the method of triangulation used in this study, aided in unearthing differences in agreement on what constitute small ruminant feeds.

Revisiting the issue of the small ruminant feeds domain, the 15 consensus items and associated salience indexes represent farmers' perceptions [33]. Pair wise ranking, although based on aggregation of feeds into categories, identified these perceptions. Focus groups based their ranking on attributes such as convenience, availability, palatability, proximity, abundance, reliability, and health risk. Thus, while natural pasture species were ranked highest for convenience (in situ grazing) and safety (no scouring), wild browse was ranked higher than crop residue for availability in all seasons, and higher than CMTS for being more abundant. Some of the reference criteria used by farmers in this study are comparable to those reported for tree fodder by Mekoya et al. [34] in Ethiopia and Thorne et al. [35] in Nepal.

Pair wise ranking scores agreed with the feed list categorization (Figure 2) and the MOFA study in 2008 [7], that natural pasture species and wild shrubs are very important in small ruminant feeding in the district. The higher score for wild browse at Kasei reflects more dependence on such feeds there. Crops had no score in all groups. According to focus groups, crops are for household food, and to feed small ruminants with crops such as maize, cassava and yam is not something a farmer would do on purpose, as long as other alternatives are available. Maize scored highest in salience analysis due to the order and high frequency of mention. Maize farmers, however, use maize grains in small amounts to tame their stock. They argued that feeding roasted salted maize grains from the palm to animals that are new to the flock aided the animals' recognition of the farmer to the extent that the animal would subsequently follow the farmer around. Both men and women ranked CMTS and crop by-products higher than crop residue due to proximity, as the former are available at the homestead and township, whereas the latter are available mainly on croplands, which are mostly distant. Few crop residues, e.g. banana and plantain leaves, can be obtained from home gardens in small quantities.

### The small ruminant feed system

All fed feeds that the 36 households agreed on as most used were also members of the free list consensus model. This shows agreement between data obtained at community and household levels.

The greater involvement [13] of women in household food preparation could explain the higher salience of crop peels for them compared with men. This, however, contradicts the freelisting result. As earlier mentioned, freelisting established people's perceptions of the small ruminant feeds domain [33] as compared to feeds fed, which indicated preference and use. For the same reason, most matrilineal groups are from Kasei and use yams to prepare fufu, the favourite dish, which increases yam peel availability. Kasei farmers also depend on the many stands of *Ficus umbellata *in the township for feed. Thus, farmers will feed what is locally and readily available.

The low frequency of mention of natural pasture species as fed feed is attributed to the *in situ *grazing of such species, and not an indication of being less important, which issue was discussed in the previous section.

Considering how the feed system was defined in this study, the major access groups, based on how small ruminants access feed, which are described in Additional file 2, could be regarded as the major feed systems at household level. This gives an indication of the flexibility of stock movement within the household farm system, which could be influenced by location, as is evident from Landscape Niche Calendars, or by household factors such as labour availability for fodder collection, which is an issue for empirical verification. The existence of new and bigger groups, 'leafyhome', 'leafyfarm' and 'cropnbyprod', gives evidence of the feed system at village level based on feed type and source. The latter grouping could facilitate the identification of possible small ruminant feed interventions at village level, based on which major feeds are used and their sources. The quantity and quality of these feeds were not the subject of this study, but empirical literature shows a wide variation in the crude protein (CP) content of the major feeds fed by farmers in this study. For feeds obtained from farmlands (Leafyfarm), CP ranges of 9-16%, 15-21%, and 20 - 29% were reported for *M. discoidea*, *P. erinaceus *and cassava leaves, respectively [36-39]. Values of 9 - 11% and 8 - 10% have been reported for banana leaves and mango leaves (Leafyhome), respectively [38,40-42], and 4 - 7%, 5-11%, and 7 - 11% for cassava peels, yam peels, and plantain peels (Cropnbyprod), respectively [36,43,44]. There is a need to investigate efficient ways of combining these feeds as supplements to natural pasture, or as sole feeds for small ruminants in both cropping and non-cropping seasons.

### Relationships between the small ruminant feed system, small ruminant performance and farmers' socio-economic circumstances

A significantly higher proportion of patrilineal households belonged to tethering access groups. With more tethering at Kasei, it can thus be inferred that patrilineal groups at Kasei tethered more than matrilineal groups. The probable explanation could be that patrilineal households are mostly migrants (settlers) and more often build on the village fringes bordering nearby farms. Sole zero grazing of *Margaritaria discoidea *and cassava leaves (Additional file 2) is more likely to occur in Kobriti, where tethering was limited. The significantly higher proportion of higher status households in the scavenging and tethering and zero grazing group (Table 5) could be due to greater labour availability for cutting feed and tethering. This needs to be confirmed with an in-depth study of how household labour conditions relate with small ruminant feeding. The fact that matrilineal heads in non-tethering access groups are significantly older could explain why less tethering was reported in matrilineal groups. Old age seems to be linked with no tethering because of the work involved, as was evident from the persistent failure by households with aged heads to restrain stock for weighing during the study.

Among the 35 households that belonged to either or both leafy groups, the significantly higher proportion of 'leafyhome' households in Kasei could be explained by greater dependence on such feeds by tethering patrilineal households or old non-tethering matrilineal households.

The pre-weaning ADGs obtained in this study are lower than the 60 g and 54 g reported by Mensah [18] for research station and traditionally managed goats, respectively, but comparable to the 29.0 - 39.6 reported by Tuah et al. [45] for the same breed. The higher incidence of tethering could have contributed to the lower ADG in patrilineal households, but, for female headed and Kobriti households, this cannot be confirmed due to the small numbers of kids involved in the calculations. However, the study showed that female headed households depended more on crop peels with low crude protein contents ranging from 4-11% [36,43,44]. Male headed households depended more on leafy feeds with higher crude protein contents of 9-29% [36-39]. The difference in feeds fed could contribute to differences in kid ADG between male and female headed households. One key informant argued that animals should perform better when men were present in the household, as men have more time to spend on their animals since they are not occupied with household chores like women are. Although this assertion seems to support the differences in ADG between male and female headed households, there is the need to investigate the time spent by each household type, sex and age group in small ruminant rearing. Prolificacy in this study is comparable to the 185.5% obtained by Tuah et al. [45] for the same breed.

Comparing the pre-weaning ADGs obtained in this study with those obtained by Mensah [18], it could be inferred that there is still scope for improvement, which we propose could be achieved through feeding, given the insights gained from this study on the feed system.

## Conclusions

This study has documented as many as 175 feeds that small ruminants eat, most of which fall into the category of crop residue, natural pasture, and wild browse. The feeds form a wide feed resource base for investigation into small ruminant feeds in the Ejura-Sekyedumasi District and other parts of the transitional zone. Farmers however agreed on only 15 feeds as the most important. Future interventions into small ruminant feeds in the short term should focus first on these 15 feeds, as they are more likely to be used by farmers, based on factors such as convenience, availability, proximity, abundance, and reliability. For instance, the *in situ *grazing of natural pasture species by small ruminants is convenient for farmers, since it saves them time. As a medium term measure, it can be recommended that village elders and their communities, with government support, create non-cropping zones within a reasonable radius of village fringes, to facilitate easier access to grazing land. Regulations may have to be put in place to organise the sustainable use of such areas, and a compensation package instituted for affected land owners.

Thirty three feeds, four of which are considered more salient for farmers, are fed by farmers themselves, namely, maize grains, cassava peels, *Margaritaria discoidea *and *Ficus umbellata*. Maize grains are used in small quantities to tame stock, which is considered very important under the extensive conditions pertaining in smallholder systems. For the protein-rich leafy feeds, *Margaritaria discoidea *and *Ficus umbellata*, it is recommended that agronomic research to increase their production is recommended, coupled with nutritional investigations for their judicious combination with cassava peels and other low-protein feeds.

Feed system as defined and used in the study and the simple analysis by cross-tabulation has simultaneously revealed a nested classification of how different feeds fed to small ruminants are accessed by these animals in different households and from different sources within the community.

There is some linkage of feed systems with factors such as economic status and age and sex of the household head as well as lineage and village location on one hand, and small ruminant performance on the other.

The results provide ample room for tailor-made feed research in particular feed systems, households, or communities, to come up with innovations that would be readily adopted. For women and other labour-constrained households, such innovations should not be labour intensive. A study of household labour inputs into different feed systems is therefore recommended to guide development of future innovations. The insights gained from this study on farmers' perceptions and practices on small ruminant feeds could guide the selection and introduction of feed innovations that fit into the current feed systems to enhance adoption for higher small ruminant performance for poverty alleviation.

## List of abbreviations

MOFA: Ministry of Food and Agriculture; NGOs: Non Governmental Organizations; ANOVA: Analysis of Variance; ADG: Average daily gain; CMTS: Cultivated multipurpose trees and shrubs

## Competing interests

The authors declare that they have no competing interests.

## Authors' contributions

All the authors participated in the design of the study and SD carried out the field study and identified some of the species. All authors contributed to the compilation of the manuscript.

## Supplementary Material

Additional file 1 **Classification of freelisted plant species and the parts used as small ruminant feed**. The list of species freelisted as small ruminant feeds by 41 farmers, showing the family, scientific name, common name, and/or local name as known in some ethnic groups in the study area, and parts used as feed. Voucher specimens of species that could be identified by the lead researcher, mainly crops, common weeds, and fruit trees were not collected. Species that could not be easily identified were sent for identification at the Forestry Research Institute of Ghana, and the Ghana Herbarium at the University of Ghana. In rare cases, the farmer could not name a species but pointed it out at the backyard. Such species were either identified by the researcher or sent for identification. Three species mentioned could not be obtained for identification and are not included in the list. Click here for file

Additional file 2 **Distribution of major feeds according to major access group, source and season**. Inventory of major small ruminant feeds fed by 36 households, classified by access group (with a description of each access group), the number of households in which the feed was mentioned (frequency), and source of the feed. Click here for file

## References

[B1] Saadullah M Hossain MM Akhter S Experiences with goat project as a tool in human development: goats for poor women in Bangladesh. Integrated farming in human development Proceedings of a workshop held on 25-29 March, 1996 at Tune Landboskole, Denmark 1997 308 319

[B2] Kristjanson P Krishna A Radeny M Nindo W Pathways out of poverty in Western Kenya and the role of livestock. PPLPI Working Paper No. 14 2004 http://www.fao.org/ag/againfo/programmes/en/pplpi/docarc/wp14.pdf Last accessed on 28^th ^February, 2010

[B3] Peacock C Goats - a pathway out of poverty Small Rumin Res 2005 60 179 186 10.1016/j.smallrumres.2005.06.011

[B4] Dossa LH Rischkowsky B Birner R Wollny C Socio-economic determinants of keeping goats and sheep by rural people in southern Benin Agric Human Values 2008 25 581 592 10.1007/s10460-008-9138-9

[B5] Anonymous Ghana poverty reduction strategy 2003-2005. An agenda for growth and prosperity. Analysis and policy statement 2003 1 http://siteresources.worldbank.org/GHANAEXTN/Resources/Ghana_PRSP.pdf Last accessed on 28^th ^February, 2010

[B6] Anonymous Areas with comparative advantage for the production of selected crops in Ghana 2002 A Compilation from the Regional and District Consultative Meeting, Ministry of Food and Agriculture (MOFA)

[B7] Anonymous Livestock Growth Trend 2008 Ministry of Food and Agriculture (MOFA) Consultancy Report

[B8] Bosman HG Ademosun AA Koper-Limbourg HAG Goat feeding practices and options for improvement in six villages in south-western Nigeria Small Rumin Res 1996 19 201 211 10.1016/0921-4488(95)00745-8

[B9] Oppong-Anane K The pasture resource FAO Grassland and Pasture Crops. Country Pasture/Forage Resources Profile. Ghana 2001 http://www.fao.org/ag/AGP/AGPC/doc/Counprof/Ghana.htm Last accessed on 28^th ^February, 2010

[B10] Gyasi E Agyepong GT Ardayfio-Schandorf E Enu-Kwesi L Nabila JS Owusuh-Benoah E Production pressure and environmental change in the forest-savannah zone of southern Ghana Glob Environ Change 1995 5 4 355 366 10.1016/0959-3780(95)00070-5

[B11] Grenier L Working with indigenous knowledge. A guide for researchers 1998 International Development Research Centre http://www.idrc.ca/en/ev-9310-201-1-DO_TOPIC.html Last accessed on 28^th ^February, 2010

[B12] Sillitoe P The development of indigenous knowledge. A new applied anthropology Currt Anthropol 1998 39 2 223 252 10.1086/204722

[B13] Ayantunde AA Briejer M Hiernaux P Udo HMJ Tabo R Botanical knowledge and its differentiation by age, gender and ethnicity in Southwestern Niger Hum Ecol 2008 36 881 889 10.1007/s10745-008-9200-7

[B14] Innes RR A Manual of Ghana Grasses 1977 Land Resources Division, Min. of Overseas Development, Surbiton, Surrey, U.K

[B15] Abbiw DK Useful plants of Ghana: West African uses of wild and cultivated plants 1990 Intermediate Technology Publications, London

[B16] Dokosi OB Herbs of Ghana 1998 Council for Scientific and Industrial Research: Ghana Universities Press

[B17] London JC Weniger JH Investigations into traditionally managed Djallonke-sheep production in the humid and sub humid zones of Asante, Ghana. I. The natural condition and the agricultural resources of the area J Anim Breed Genet 1994 111 314 336 10.1111/j.1439-0388.1994.tb00471.x 21395783

[B18] Mensah BA Productivity of traditionally-managed small ruminants in the Ejura Sub-district of the Ashanti Region of Ghana MPhil thesis 1996 Kwame Nkrumah University of Science and Technology, Kumasi, Department of Animal Science

[B19] Howard PL Howard PL Women and the plant world: an exploration Women & Plants. Gender Relations in Biodiversity Management and Conservation 2003 London and New York: Zed Press and Palgrave Macmillan 1 48

[B20] Simpson BM Gender and the social differentiation of local knowledge IK Monitor 1994 2 3 http://www.iss.nl/ikdm/IKDM/IKDM/2-3/articles/simpson.html Last accessed on 28^th ^February, 2010

[B21] Rangnekar SD Studies on the knowledge of rural women regarding local feed resources and feeding systems developed for livestock Livestock Research for Rural Development 1994 6 1 http://www.lrrd.org/lrrd6/1/india.htm Last accessed on 28^th ^February, 2010

[B22] Codjoe SNA Migrant versus indigenous farmers. An analysis of factors affecting agricultural land use in the transitional agro-ecological zone of Ghana, 1984-2000 Geogr Tidsskr 2006 106 1 103 113

[B23] Amanor KS Pabi O Space, time, rhetoric and agricultural change in the transition zone of Ghana Hum Ecol 2007 35 51 67 10.1007/s10745-006-9081-6

[B24] Punch KF Introduction to Social Research. Quantitative and Qualitative Approaches 2005 2 Sage Publications

[B25] Bernard HR Research Methods in Anthropology. Qualitative and Quantitative Approaches 2006 Altamira Press

[B26] Borgatti SP ANTHROPAC 4.0 Methods Guide Natick, MA: Analytic Technologies 1996

[B27] Howard P Smith E Leaving two thirds out of development: female headed households and common property resources in the highlands of Tigray, Ethiopia LSP Working Paper 40. FAO, Rome 2006 ftp://ftp.fao.org/docrep/fao/009/ah624e/ah624e00.pdf Last accessed on 28^th ^February, 2010

[B28] Borgatti SP ANTHROPAC 4.0. Natick, MA: Analytic Technologies 1996

[B29] Smith JJ Borgatti SP Salience counts - so does accuracy: correcting and updating a measure for free-list item salience J Linguist Anthropol 1998 7 2 208 209 http://www3.interscience.wiley.com/cgi-bin/fulltext/120181908/PDFSTART Last accessed on 28^th ^February, 2010 10.1525/jlin.1997.7.2.208

[B30] Romney AK Weller SC Batchelder WH Culture as consensus - a theory of culture and informant accuracy Am Anthropol 1986 88 313 338 10.1525/aa.1986.88.2.02a00020

[B31] Schrauf RW Sanchez J Using freelisting to identify, assess, and characterize age differences in shared cultural domains J Gerontol 2008 63B 6 S385 S393 10.1093/geronb/63.6.s385 19092048

[B32] Field A Discovering statistics using SPSS (and sex, drugs and rock 'n'roll) 2005 London: Sage

[B33] Borgatti S Schensul EJJ, LeCompte MD, Nastasi BK, Borgatti SP Elicitation techniques for cultural domain analysis Enhanced Ethnographic Methods: Audiovisual Techniques, Focused group interviews, and Elicitation Techniques. Ethnographer Toolkit 1999 CA Walnut Creek: Altamira Press 115 151

[B34] Mekoya A Oosting SJ Fernandez-Rivera S Zijpp AJ Van der Multipurpose fodder trees in the Ethiopian highlands: farmers' preference and relationship of indigenous knowledge of feed value with laboratory indicators Agric Syst 2008 96 184 194 10.1016/j.agsy.2007.08.001

[B35] Thorne PJ Subba DB Walker DH Thapa B Wood CD Sinclair FL The basis of indigenous knowledge of tree fodder quality and its implications for improving the use of tree fodder in developing countries Anim Feed Sci Technol 1999 81 119 131 10.1016/S0377-8401(99)00048-6

[B36] ILRI. Sub-Saharan Africa feed composition database. ILRI lab data. The CGIAR Systemwide Livestock Program http://www.vslp.org/ssafeed/variables.asp Last accessed on 28^th ^February, 2010

[B37] Msangi RBR Hardesty LH Forage value of native and introduced browse species in Tanzania J Range Manag 1993 46 5 410 415 10.2307/4002658

[B38] Keir B Nguyen VL Preston TR Orskov ER Nutritive value of leaves from tropical trees and shrubs: 1 In *vitro gas *production and *in sacco *rumen degradability Livest Res Rural Dev 1997 9 4 http://www.lrrd.org/lrrd9/4/bren941.htm Last accessed on 28^th ^February, 2010

[B39] Osakwe II Steingass H Drochner W The chemical composition of *Phyllanthus discoideus *and its effect on the ruminal ammonia and volatile fatty acid concentration when fed to WAD sheep Arch Anim Nutr 2000 53 191 205 10.1080/17450390009381946 10849871

[B40] Le Houérou HN Le Houérou HN Chemical composition and nutritive value of browse in tropical West Africa Proceedings of the International Symposium on Browse in Africa: 8-12 April 1980 Addis Ababa (Ethiopia). International Livestock Centre for Africa 1980 International Livestock Center for Africa: Addis Ababa 261 289

[B41] Aschfalk A Steingass H Müller W Drochner W Acceptance and digestibility of some selected browse feeds with varying tannin content as supplements in sheep nutrition in West Africa J Vet Med A 2000 47 513 524 10.1046/j.1439-0442.2000.00313.x 11246480

[B42] Katongole CB Bareeba FB Sabiiti EN Ledin I Nutritional characterization of some tropical urban market crop wastes Anim Feed Sci Technol 2008 142 275 291 10.1016/j.anifeedsci.2007.09.002

[B43] Tuah AK Dzowela BH, Said AN Utilization of agricultural by-products for village and commercial production of sheep rations in Ghana Proceedings of the First Joint PANESA AND ARNAB Workshop on utilization of research results on forage and agricultural by-product materials as animal feed resources in Africa: 5-9 Dec. 1988 1990 Lilongwe, Malawi: Agrat Wendem - Agenehu, Kategile JA. ILCA 57 69

[B44] Smith OB Idowu OA Asaolu VO Odunlami O Comparative rumen degradability of forages, browse, crop residues and agricultural by-products Livest Res Rural Dev 1991 3 2 http://www.lrrd.org/lrrd3/2/smith.htm Last accessed on 28^th ^February, 2010

[B45] Tuah AK Buadu MK Obese FY Brew K Rey B, Lebbie SHB, Reynolds L The performance, potentials and limitations of the West African Dwarf goat for meat production in the forest belt of Ghana Proceedings of the 1st Biennial Conference of the African Small Ruminant Research Network on Small Ruminant Research and Development in Africa: 10-14 December 1990; 1990 Nairobi, Kenya. ILRAD 435 441

