# Supplementary material for: Small ruminant feed systems: perceptions and practices in the transitional zone of Ghana
Source: J Ethnobiol Ethnomed. 2010 Mar 19;6:11. doi: 10.1186/1746-4269-6-11 (PMC3224954; doi:10.1186/1746-4269-6-11)
Supplement: Additional file 1 — Classification of freelisted plant species and the parts used as small ruminant feed. The list of species freelisted as small ruminant feeds by 41 farmers, showing the family, scientific name, common name, and/or local name as known in some ethnic groups in the study area, and parts used as feed. Voucher specimens of species that could be identified by the lead researcher, mainly crops, common weeds, and fruit trees were not collected. Species that could not be easily identified were sent for identification at the Forestry Research Institute of Ghana, and the Ghana Herbarium at the University of Ghana. In rare cases, the farmer could not name a species but pointed it out at the backyard. Such species were either identified by the researcher or sent for identification. Three species mentioned could not be obtained for identification and are not included in the list. [file 1746-4269-6-11-S1.PDF]

Additional file 1. Classification of freelisted plant species and the parts used as small ruminant feed

Family	***Scientific name***	***Common name***	***Local name***	***Parts used as feed***
Acanthaceae	*Dyschoriste perrottetii* (Nees) Kuntze	-	-	Leaves
	*Justicia flava (*Forsk.) Vahl.	Yellow justicia	Ntumunum (Twi)	Leaves
Amaranthaceae	*Amaranthus hybridus* L.	Smooth amaranth	Alayafu (Hausa)	Leaves
	Amaranthus spinosus L.	Prickly amaranth	Srahansoe (Twi)	Leaves
	Cyathula prostrata (L.) Blume	-	Mpupuaa (Twi)	Leaves
Anacardiaceae	*Anacardium occidentale* L.	Cashew	Atea (Twi)	Leaves
	Mangifera indica L.	Mango	Mango	Leaves, fruits, peels
	Spondias mombin L.	Yellow mombin	Atoa (twi)	Leaves
Annonaceae	*Annona senegalensis* Pers.	African custard-apple	Aboboma (Twi)	Leaves
	Annona senegalensis Pers. subsp. senegalensis Pers.	African custard-apple	Karga (Moshi)	Leaves
Apiaceae	*Daucus carota* L.	Carrot	-	Leaves, tubers
Apocynaceae	*Holarrhena floribunda (*G. Don) Dur. et Schinz	False rubber tree	Sese (Twi)	Leaves
Araceae	*Xanthosoma spp* (L.) Schott	Cocoyam	Mankani (Twi)	Leaves
Arecaceae	*Cocos nucifera* L.	Coconut	Kube (Twi)	Leaves
	Elaeis guineensis Jacq.	Oil palm	Abe (Twi)	Leaves
Asteraceae	*Acanthospermum hispidum* DC.	Bristly starbur	Srahansoe (Twi)	Leaves
	Bidens pilosa L.	Spanish needles	Gyinantwi (Twi)	Leaves
	*Chromolaena odorata (*L.) King and Robinson	Siam weed	Akyeampong (Twi)	Leaves
	Synedrella nodiflora Gaertn.	Nodeweed	Yaa Asantewa (Twi)	Leaves
	Tridax procumbens L.	Coat buttons	-	Leaves
	Vernonia amygdalina (Schreber)	Bitter leaf	Awonyono (Twi)	Leaves
Bignoniaceae	*Crescentia cujete* L.	Callabash tree	Koraa (Twi)	Leaves
Brassicaceae or Cruciferae	*Brassica oleracea* var. *capitata*	Cabbage	-	Leaves
Caesalpiniaceae	*Tamarindus indica* L.	Tamarind	Pusiga (Moshi)	Leaves
Caricaceae	*Carica papaya* L.	Pawpaw	Brofre (Twi)	Leaves, fruits, peels
Combretaceae	*Anogeissus leiocarpus* (DC.) Guill. & Perr	-	Kane (Twi)	Leaves
	Terminalia catappa L.	Indian almond	Abrofo nkate (Twi)	Leaves

	Terminalia glaucescens (Hochst.)	-	Enwo (Twi)	Leaves
Commelinaceae	*Commelina diffusa* Burm. F.	Commelina	Onyamebewunamawu (Twi)	Leaves
Convulvulaceae	*Ipomoea batatas* L.	Sweet potato	-	Leaves, tubers
Cucurbitaceae	*Citrullus lanatus*L.	-	Wrewre (Twi)	Leaves, seeds, husk
	Citrullus colocynthis (L) Schrad.	Water melon	-	Leaves, fruits, peels
	Cucumeropsis manii Naud.	-	Akatoa (Twi); agushi	Leaves, seeds, husk
	Cucurbita pepo L.	Pumpkin	Efere (Twi)	Leaves
	Momordica charantia L.	African cucumber	Nyanya (Twi)	Leaves
Cyperaceae	*Cyperus rotundus* L.	Nut grass	Hyiamenonnum (Twi)	Leaves
Dioscoreaceae	*Dioscorea sp*	Yam	Bayere (Twi)	Leaves, tubers, peels
Ebenaceae	*Diospyros mespiliformis* (Hochst*)*	African ebony	Gaaka (Moshi)	Leaves
Erythroxylaceae	*Erythroxylum emarginatum* Thonn.	-	Kokoowa (Twi)	Leaves
Euphorbiaceae	*Bridelia micrantha* (Hochst) Baille.	-	Badie (Twi)	Leaves
	*Alchornea cordifolia (*Schumm. and Thonn.) Muell. Arg.	-	Gyamma (Twi)	Leaves
	Euphorbia hirta L.	Asthma weed	Kakaweadwe (Twi)	Leaves
	Mallotus oppositifolius (Muel & Arg)	-	Nyanyanforowa; Satadua (Twi)	Leaves
	Manihot esculenta (Crantz.)	Cassava	Bankye (Twi)	Leaves, peels, tubers
	Margaritaria discoidea (Baill.) G.L.Webster	-	Papea	Leaves
	Phyllanthus niruri L.	-	Awommaguwakyi (Twi)	Leaves
Fabaceae	*Arachis hypogaea* L.	Groundnut	Nkate (Twi)	Leaves, pods, seeds
	Cajanus cajan (L.) Millsp.	Pigeon pea	Akye	Leaves
	Centrosema pubescens Benth	Centro	Ananse ntrumu nhoma	Leaves
	Glycine max (Wild)	Soya bean	-	Leaves, pods, seeds
	Leucaena leucocephala (Lam.) de Wit	Leucena	-	Leaves
	Parkia biglobosa (Jacq.) Willd.	African locust bean	Dawadawa	Leaves, fruits, seeds
	Pterocarpus erinaceus Poir.	-	Krayie (Twi)	Leaves
	Vigna unguiculata (L.) Walp	Cowpea	Waakye (Hausa)	Leaves, pods, seeds

	Voandzeia subterranean (*Vigna subterranea* (L.) Verdc.)	Bambara bean	-	Leaves, seeds
Lauraceae	*Persea americana* (Mill.)	Avocado	Paya (Twi)	Leaves, peels
Loranthaceae	*Tapinanthus bangwensis* (Engl.& K Krause) Danser	Mistletoe	Nyankunuru (Twi)	Leaves
Malvaceae	*Adansonia digitata* (L.) Medic.	Baobab	Kuka (Hausa)	Leaves
	*Ceiba pentandra (*L.) Gaertn.	Silk cotton tree	Onyina (Twi)	Leaves
	Abelmoschus esculentus (L.) Moench	Okra	Nkuruma (Twi)	Leaves, fruits
	Bombax buonopozense Beauv.	Gold Coast bombax	Akata (Twi)	Leaves
	*Cola nitida (*Vent.) Schott and Endl.	Cola	Bese (Twi)	Leaves
	Corchorus capsularis	White jute		Leaves
	Hibiscus cannabinus L.	Kenaf		Leaves
	Hibiscus sabdariffa L.	Red sorrel	Suule (Hausa)	Leaves, seeds
	Sida acuta Burm. f.	Common wire weed	Obraneatuata (Twi)	Leaves
Marantaceae	*Marantochloa cuspsidata* (Rosc.) Milne-Redhead	-	Aworomo (Twi)	Leaves
Meliaceae	*Azadirachta indica* (A. Juss)	Neem		Leaves
	Khaya senegalensis (Desr.) A.Juss.	African mahogany	Koga (Moshi)	Leaves
Mimosaceae	*Albizia ferruginea (*Guill. and Perr.) Benth.	-	Awiemfosemina (Twi)	Leaves
Moraceae	*Ficus umbellata* Vahl.	-	Mangyedua (Twi)	Leaves
	Ficus exasperata Vahl.	Sandpaper tree	Nyankyeren	Leaves
	Ficus spp	-		Leaves
	Ficus sur Forssk.	-	Odoma	Leaves
	Ficus sycomorus L.	Small leaf Sycamore fig	Kankanga	Leaves
Moringaceae	*Moringa oleifera* Lam.	Moringa	Arizanteega (Moshi)	Leaves
Musaceae	*Musa paradisiaca* L.	Plantain	Brode (Twi)	Leaves, fingers, peels, sucker
	Musa sapientum L.	Banana	Kwadu (Twi)	Leaves, fruits, peels
Nyctaginaceae	*Boerhavia diffusa* L.	Hogweed	Nkwadaa bayere (Twi)	Leaves
Poaceae	*Andropogon gayanus* Kunth var. Gayanus Hack.	Gamba grass	Foroforo (Twi)	Leaves

	Axonopus compressus (Sw.) Beauv.	Broadleaf carpet grass	-	Leaves
	Chloris pilosa Schum.		-	Leaves
	Cymbopogon citratus Spreng.	Lemongrass	-	Leaves
	*Cynodon dactylon (*L.) Pers.	Bermuda grass	-	Leaves
	Dactyloctenium aegyptium (L.) P. Beauv	Crowfoot grass	-	Leaves
	Digitaria insularis (Lock)	Sourgrass	Rawlings	Leaves
	*Eleucine indica (*L.) Gaertn.		-	Leaves
	Imperata cylindrica (Linn.) P. Beauv. var. *thunberigii* (Retz.) Dur. and Schinz.	Spear grass	Fan	Leaves
	Oryza glaberrima	Rice	Emo (Twi)	Leaves, paddy, straw
	Panicum maximum Jacq.	Guinea grass	-	Leaves
	Paspalum conjugatum Berg.	Green grass	Nsonwea (Twi)	Leaves
	Pennisetum glaucum (L.) R. Br. 1810	Millet	Ewio (Twi)	Leaves, grains, offal, mash
	Pennisetum pedicellatum Trin.	Kyasuma grass	-	Leaves
	Pennisetum purpureum Schum.	Elephant grass	Hwidie (Twi)	Leaves
	Rottboellia cochinchinensis (Lour.) Clayton	Itch grass	Kyenkyema (Twi)	Leaves
	Saccharum officinarum L.	Sugar cane	Ahwedee (Twi)	Leaves
	Sorghum bicolor (L.) Moench	Guinea corn	Atokoo (Twi)	Leaves, grains, mash
	Sporobolus pyramidalis P. Beauv	Rat's tail grass	Abirekyie abogyese (Twi)	Leaves
	Zea mays L.	Maize	Aburoo (Twi)	Leaves, grains, husk, cobs, straw, offal
Portulacaceae	*Portulaca oleracea* L.	Purslane	Adwere (Twi)	Leaves
	Portulaca quadrifida L.	Small-leaved purslane	Asaaseneabo (Twi)	Leaves
	*Talinum triangulare (*Jacq.) Willd.	waterleaf	Bokoboko (Twi)	Leaves
Rubiaceae	*Nuclea latifolia* Sm.	-	Awodi (Atakwame)	Leaves
Rutaceae	*Citrus limon* (L.) Burm.f.	Lemon	-	Leaves
	Citrus sinensis (L.) Osbeck	Sweet orange	Ankaa (Twi)	Leaves, peels
	Zanthoxylum zanthoxyloides (Lam.) Zepernick & Timler	-	Yoreyore (Konkomba); Okanto (Twi)	Leaves

Sapindaceae	*Blighia sapida* Konig	Akee apple	Akye (Twi)	Leaves
	Paullinia pinnata L.	-	Toantin (Twi)	Leaves
Sapotaceae	*Vitellaria paradoxa* C. F. Gaertn.	Shea butter tree	Krankun (Twi)	Leaves, nuts
Solanaceae	*Capsicum frutescens* L.	Red pepper	Mako (Twi)	Leaves
	Physalis angulata L.	-	Totototo (Twi)	Leaves
	Solanum aethiopicum L.	Garden eggs	Ntrowa (Twi)	Leaves, fruits
	Lycopersicum esculentum Mill.	Tomato	Ntoos (Twi)	Leaves, fruits
	Solanum torvum (Sw)	Turkey berry	Kwahu Nsusuaa (Twi)	Leaves
Tiliaceae	*Corchorus olitorius* L.	Tossa jute	Ayoyo	Leaves
	Grewia lasiodiscus (K.Schum)	-	Yologa (Moshi)	Leaves
Verbenaceae	*Vitex doniana* Sweet	Black plum	Aadaga (Moshi)	Leaves
	Gmelina arborea (Roxb)	-	Galama	Leaves
	Tectona grandis Linn F.	Teak	-	Leaves
Vitaceae (Ampelidaceae)	*Cissus populnea* Guill and Perr.	-	Asakono (Twi)	Leaves
