# Supplementary material for: Small ruminant feed systems: perceptions and practices in the transitional zone of Ghana
Source: J Ethnobiol Ethnomed. 2010 Mar 19;6:11. doi: 10.1186/1746-4269-6-11 (PMC3224954; doi:10.1186/1746-4269-6-11)
Supplement: Additional file 2 — Distribution of major feeds according to major access group, source and season. Inventory of major small ruminant feeds fed by 36 households, classified by access group (with a description of each access group), the number of households in which the feed was mentioned (frequency), and source of the feed. [file 1746-4269-6-11-S2.PDF]

## Additional file 2. Distribution of major feeds according to major access group, source and season

| *Access group (frequency of cases)* | *Access group description* | *Important feeds (freuency)* | *Source* | | | | | | | | | | | | | | | | | | | | |
|---|---|---|---|---|---|---|---|---|---|---|---|---|---|---|---|---|---|---|---|---|---|---|---|
| | | | *Public land* | | | *Own land* | | | *Other's land* | | | *Home garden* | | | *Own kitchen* | | | *Other's kitchen* | | | *Own production* | | |
| | | | *Season* | | | | | | | | | | | | | | | | | | | | |
| | | | cs | as | ac | cs | as | ac | cs | as | ac | cs | as | ac | cs | as | ac | cs | as | ac | cs | as | ac |
| 1 (37) | scavenging & tethering & zero grazing | *Ficus umbellata* (10) | 1 | 5 | 0 | 2 | 8 | 0 | 2 | 6 | 0 | 2 | 7 | 0 | | | | | | | 2 | 3 | 0 |
| | | Banana leaves (10) | 0 | 6 | 0 | 0 | 9 | 0 | 0 | 8 | 0 | 0 | 10 | 0 | | | | | | | 0 | 6 | 0 |
| | | Mango leaves (6) | 1 | 4 | 0 | 1 | 5 | 0 | 1 | 2 | 0 | 0 | 3 | 0 | | | | | | | 0 | 0 | 0 |
| 2 (42) | scavenging & tethering & hand feeding | Maize grains (15) | | | | | | | | | | | | | 0 | 0 | 0 | 0 | 0 | 0 | 0 | 10 | 5 |
| | | Cassava peels (13) | | | | | | | | | | | | | 0 | 13 | 0 | 0 | 6 | 0 | 0 | 11 | 0 |
| | | Yam peels (9) | | | | | | | | | | | | | 0 | 9 | 0 | 0 | 2 | 0 | 0 | 4 | 0 |
| 3 (27) | scavenging & zero grazing, not tethering | *Ficus umbellata* (6) | 0 | 3 | 1 | 0 | 5 | 1 | 0 | 5 | 1 | 0 | 4 | 1 | | | | | | | 0 | 2 | 0 |
| 4 (59) | scavenging & hand feeding, not tethering | Maize grains (17) | | | | | | | | | | | | | 0 | 0 | 0 | 0 | 0 | 0 | 0 | 11 | 6 |
| | | Cassava peels (18) | | | | | | | | | | | | | 0 | 17 | 1 | 0 | 6 | 1 | 0 | 15 | 1 |
| | | Yam peels (8) | | | | | | | | | | | | | 0 | 7 | 1 | 0 | 1 | 0 | 0 | 7 | 0 |
| | | Plantain peels (6) | | | | | | | | | | | | | 2 | 4 | 0 | 1 | 1 | 0 | 0 | 2 | 0 |

| | | | | | | | | | | | | | | |
|---|---|---|---|---|---|---|---|---|---|---|---|---|---|---|
| 5 (28) | tethering & zero grazing, not scavenging | *Margaritaria discoidea* (13) | 9 | 4 | 0 | 9 | 3 | 0 | 9 | 4 | 0 | | | |
| | | *Pterocarpus erinaceus* (6) | 5 | 1 | 0 | 5 | 1 | 0 | 5 | 1 | | | | |
| 6 (29) | zero grazing only | *Margaritaria discoidea* (8) | 0 | 7 | 0 | 1 | 7 | 0 | 1 | 7 | 0 | 0 | 0 | 0 |
| | | Cassava leaves (7) | 0 | 0 | 0 | 6 | 1 | 0 | 0 | 0 | 0 | 6 | 1 | 0 |
| cs = cropping season; as = all seasons; ac = after cropping season | | | | | | | | | | | | | | |
